# Vaccine Adverse Events Following COVID-19 Vaccination with Inactivated Vaccines in Zimbabwe

**DOI:** 10.3390/vaccines10101767

**Published:** 2022-10-21

**Authors:** Azure Tariro Makadzange, Patricia Gundidza, Charles Lau, Norest Beta, Nellie Myburgh, Nyasha Elose, Wilmot James, Lawrence Stanberry, Chiratidzo Ndhlovu

**Affiliations:** 1 Charles River Medical Group, 155 King George Avenue, Avondale, Harare, Zimbabwe; 2 RTI International, 3040 East Cornwallis Road, Research Triangle Park, Research Triangle, NC 27709, USA; 3 Wits Vaccines & Infectious Diseases Analytics (VIDA) Research Unit, Faculty of Health Sciences, University of the Witwatersrand, Johannesburg 2000, South Africa; 4 Institute for Social and Economic Research and Policy, Columbia University, IAB 118th Street, New York, NY 10025, USA; 5 Vaccine Information Network, Columbia University, 533 W 218th Street, New York, NY 10032, USA; 6 Department of Pediatrics, Vagelos College of Physicians and Surgeons, Columbia University, New York, NY 10032, USA; 7 Internal Medicine Unit, Department of Primary Health Care Sciences, Faculty of Medicine and Health Sciences, University of Zimbabwe, Avondale, Harare P.O. Box A178, Zimbabwe

**Keywords:** vaccine adverse events, COVID-19 vaccination, vaccine hesitancy, inactivated COVID-19 vaccine, Sinopharm vaccine, Sinovac vaccine, whole attenuated vaccine

## Abstract

Vaccination is one of the most effective methods for preventing morbidity and mortality from COVID-19. Vaccine hesitancy has led to a decrease in vaccine uptake; driven by misinformation, fear, and misperceptions of vaccine safety. Whole inactivated vaccines have been used in one-fifth of the vaccine recipients in Africa, however there are limited real-world data on their safety. We evaluated the reported adverse events and factors associated with reported adverse events following vaccination with whole inactivated COVID-19 vaccines-BBiBP-CorV (Sinopharm) and CoronaVac (Sinovac). A quantitative survey evaluating attitudes and adverse events from vaccination was administered to 1016 adults presenting at vaccination centers. Two follow-up telephone interviews were conducted to determine adverse events after the first and second vaccination dose. Overall, the vaccine was well tolerated; 26.0% and 14.4% reported adverse events after the first and second dose, respectively. The most frequent local and systemic adverse events were pain at the injection site and headaches, respectively. Most symptoms were mild, and no participants required hospitalization. Participants who perceived COVID-19 vaccines as safe or had a personal COVID-19 experience were significantly less likely to report adverse events. Our findings provide data on the safety and tolerability of whole inactivated COVID-19 vaccines in an African population, providing the necessary data to create effective strategies to increase vaccination and support vaccination campaigns.

## 1. Introduction

The SARS-CoV-2 virus has had a significant impact on global morbidity and mortality. As of early August 2022, there have been over 583 million cases and 6.4 million reported deaths [1] with significant pandemic-related excess mortality [2]. Rapid development and deployment of vaccines was critical for enabling societies to gradually return to normalcy [3]. Despite the success of vaccines in reducing morbidity and mortality, vaccine uptake has remained poor in many parts of the world. In Africa, only 66% of doses received have been administered and only 27% of the population has received at least one dose [4]. The low coverage may be driven by vaccine hesitancy [5]. Perceived vaccine safety has been identified as an important factor in driving vaccine confidence and uptake [6,7].

Whole inactivated vaccines such as BBiBP-CorV (Sinopharm) and CoronaVac (also known as Sinovac) have been important components of the public health response to SARS-CoV-2. The vaccines have been used globally and account for 22% of the vaccines administered in Africa [4]. The vaccines were developed using traditional manufacturing techniques and adjuvants making it easier for local regulators to evaluate them, and the vaccines have similar cold-chain requirements to the other vaccines used in the Early Programs in Immunization (EPI).

The Sinopharm vaccine is administered as an intramuscular injection on a two-dose schedule. A phase 1/2 trial conducted in China that enrolled 320 participants and included up to 10µg doses demonstrated that the vaccines were immunogenic and well tolerated. The most common adverse events were injection-site pain and fever; all adverse reactions occurred within the first 7 days after dosing, were mild, self-limiting and did not require treatment [8]. A second Chinese Phase 1/2 study that enrolled 192 participants in Phase 1 and 448 in Phase 2 reported at least one adverse event in 23% of participants [9]. Adverse events were slightly more common in younger individuals than those above the age of 60 years. Most adverse events were mild with injection-site pain and fever being the most common adverse events [9]. In a phase 3 study of both the Sinopharm and Sinovac vaccines that enrolled 40,382 participants in UAE and Bahrain, the inactivated vaccines were well tolerated with a reported adverse events rate similar between the vaccine groups and alum containing placebo [10]. The most common adverse event was injection-site pain and headache [10]. In a survey of 1080 adults conducted in the UAE after the roll-out of the vaccine, the reported adverse events were mild and consisted mostly of injection-site pain, fatigue and headaches [11]. Overall, the vaccines have been well tolerated [12] with rare case reports of bilateral anterior uveitis [13].

The Sinopharm vaccine has been used in 14% of the vaccine doses administered in Africa and is the primary vaccine that has been used in Zimbabwe [4]. However, there is limited real-world data on COVID-19 vaccine adverse events following vaccination with whole inactivated vaccines in African populations. We conducted a survey among adults in Harare, Zimbabwe, to assess adverse events following first and second dose of inactivated vaccines.

## 2. Materials and Methods

### 2.1. Study Design

Between 4 January 2022 and 11 February 2022, we conducted a series of quantitative surveys to evaluate attitudes to vaccination and adverse events following COVID-19 vaccination. An in-person survey was conducted on the day the participant received the first vaccine dose at the time of enrolment with follow-up telephone interviews conducted two weeks after the first and the scheduled second vaccine dose. The survey targeted individuals presenting at the selected clinics for COVID-19 vaccination for the first time. At this time, vaccination campaigns had been initiated one year before.

### 2.2. Study Sites and Sampling

Adults (age > 18 years) who were receiving their first dose of COVID-19 vaccines at 5 City of Harare clinics, including their affiliated outreach sites (Mabelreign, Avondale, Belvedere, and Dzivarasekwa), were consecutively enrolled into the study. All individuals receiving their first COVID-19 vaccine were eligible to participate. Participants with obvious cognitive impairments or who were unable to provide informed consent were excluded.

A comprehensive questionnaire was prepared as previously described [14]. The questionnaire was administered in either English or Shona. Data were collected in REDCap using the mobile application. After an interview, each participant received USD5 as compensation for participation in the in-person interview and USD1 worth of airtime at each telephone follow-up interview. The first telephone interview was conducted two weeks after the first vaccine dose and focused on social networks for disclosing vaccination status, adverse events following the first dose and attitudes and barriers to the second dose vaccination. The second telephone interview was conducted two weeks after the scheduled second dose and assessed adverse events and barriers to obtaining the second dose. A third telephone interview was conducted 12 weeks after the first dose, for participants that had not completed their second dose at the time of the second interview. The interview assessed second dose completion and associated adverse events and attitudes and barriers to receiving the second dose (Table 1). All of the study data were confidential.

### 2.3. Statistical Analysis

Data for the categorical questions were described using absolute numbers, proportions, and percentages.

Chi-squared tests were used to compare the dose adverse events’ responses for different sub-groups i.e., gender, age, education, personal COVID-19 experience, HIV status, vaccine safety and confidence in regulatory process and vaccine effectiveness. Statistical significance was assessed using p-values. The strength of investigated associations was described using adjusted odds ratios (ORs) and corresponding to 95% confidence intervals and variables with a *p*-value ≤ 0.05 remained in the final model (two-sided). Analyses were performed using IBM SPSS Statistics^®^ version 23 (IBM Corp, Armonk, NY, USA). Multicollinearity was assessed by computing the variance inflation factor (VIF). *p*-values were two-sided and 0.05 was used as the level of confidence.

### 2.4. Ethics Approvals

The protocol, consent forms and recruitment materials were reviewed and approved by the Medical Research Council of Zimbabwe (MRCZ), Joint Research Ethics Committee of Parirenyatwa Hospital and the University of Zimbabwe (JREC) and Harare City Health Departments prior to initiation of the study. The MRCZ approval number was MRCZ/A/2809. The JREC approval number was JREC/373/2021. All amendments to the protocol, consent forms and/or recruitment materials were approved by these institutional review boards before they were implemented. All of the participants provided written informed consent.

## 3. Results

### 3.1. Demographic Characteristics

A total of 1016 adults were enrolled into the study, 1013 (99.7%) received the Sinopharm vaccine and 3 (0.3%) the Sinovac vaccine. Analysis combined the data of those who received both vaccines. Of these, a total of 943 (92.8%) and 922 (90.7%) participants were successfully interviewed for the first and second follow-up interviews, respectively. We had a total of 917 (90.3%) participants completing all three interviews. The demographic characteristics of the cohort have been previously described [14]. The characteristics of the participants that completed the follow-up interviews were similar for the three interviews (Table 2). At first follow-up 487 (51.6%) and at second follow-up 482 (52.3%) participants were female. The median age was 30 years IQR (22–39). For all three interviews, approximately 12.6% of participants were people living with HIV (PLWH). The median time between follow-up interview and first vaccine dose was 17 days IQR (16–19). The median time between second vaccine dose and follow-up interview was 10 days IQR (8–13). Among study participants, 697 (68.6%) received their second dose and three of them failed to complete the second follow-up interview. A total of 694 (68.3%) participants received their second dose and completed the second follow-up interview.

Adverse events attributed to the vaccination were reported by 244 (26%) and 100 (14.4%) participants after the first and second dose, respectively. Among the study participants who experienced adverse events, 202 (21.5%) from the first dose and 80 (11.5%) from the second dose reported only one side effect. The highest number of adverse events experienced was four and reported by four (0.4%) and four (0.6%) from the first and second dose, respectively.

Local reactogenicity was described as ‘little to no swelling or redness’ by 916 (97.1%) and 670 (96.5%) after the first and second dose, respectively. Swelling and/or redness with pain but no restriction in movement was experienced by 22 (2.3%) after the first dose and another 22 (3.2%) after the second dose. Severe swelling with pain and difficulty moving was experienced by two (0.3%) participants after the second dose. Pain in the arm was experienced by 68 (7.2%) after the first dose and 14 (2%) after the second dose. There was a significant reduction, 68 (7.2%) to 14 (2.0%), *p* < 0.00001), in the number of participants who reported pain in the arm as a side effect after the second vaccination dose compared to the first.

Several systemic reactions were reported after each vaccination dose. After the first dose, the major adverse events reported were pain (7.2%), fatigue (6.3%), headaches (4.1%), nausea and vomiting (2.8%), joint pain (2.6%) and fever (2.1%). Fatigue (5.2%) and headaches (2.5%) were the major adverse events reported after the second vaccination dose, followed by pain (2.0%), fever (1.7%), nausea and vomiting (1.7%) and joint pain (1.3%). Among the 20 (2.1%) participants who reported having a fever after the first dose, the onset was within 12 h for 5 (25%), 12–24 h for 3 (15%) and more than 24 h for 12 (60%); 11 (55.0%) reported that their fever lasted for ≥ 72 h. Among the 12 (1.7%) participants who reported having a fever after the second dose the fever started within 12 h for 7 (58.3%), between 12–24 h for 1 (8.3%) and more than 24 h for 4 (33.3%) participants. In 8 (66.7%) participants the fever lasted for more than 72 h. The frequency of reported adverse events dropped following the second dose compared to the first dose (26% vs. 14.4%, *p* = 0.02) (Table 3).

The most common adverse event experienced was fatigue. There was a significant difference in experience of fatigue after the two doses, (24.2% after first dose and 36% after second dose, *p* = 0.03).

After the first dose, 33 (3.5%) reported having to miss days of work because of adverse events from the vaccine. Most, 18 (1.9%), reported missing 1–2 days, 8 (0.8%) reported missing 3–5 days, 5 (0.5%) reported missing 6–7 days and 2 (0.2%) reported missing more than a week. There were 16 (1.7%) participants who developed symptoms that required medical attention after the first dose. The primary symptom that prompted medical attention was headache in 13 (81.3%) participants after the first dose. Following the first dose, 6 (0.6%) participants reported that they tested positive for COVID-19 after vaccination; none of those that tested positive required hospitalization.

After the second dose, the number reporting that they had to miss days of work due to the vaccine dropped down to four (0.6%) participants. Two participants (0.3%) reported missing 1 day, one (0.1%) participant reported missing 3 days and one (0.1%) participant reported missing 7 days. There were 15 (2.2%) participants who developed symptoms that required medical attention after the second dose; the primary symptom that prompted medical attention was headache in 13 (86.7%). Following the second dose, 10 (1.1%) participants reported that they tested positive for COVID-19 after vaccination; seven (70%) reported having no symptoms, one (10%) reported having mild symptoms, two (20%) reported major symptoms. None of those that tested positive for COVID-19 required hospitalization.

### 3.2. Relationship of Adverse Events with Demographic Variables

**Figure 1:**
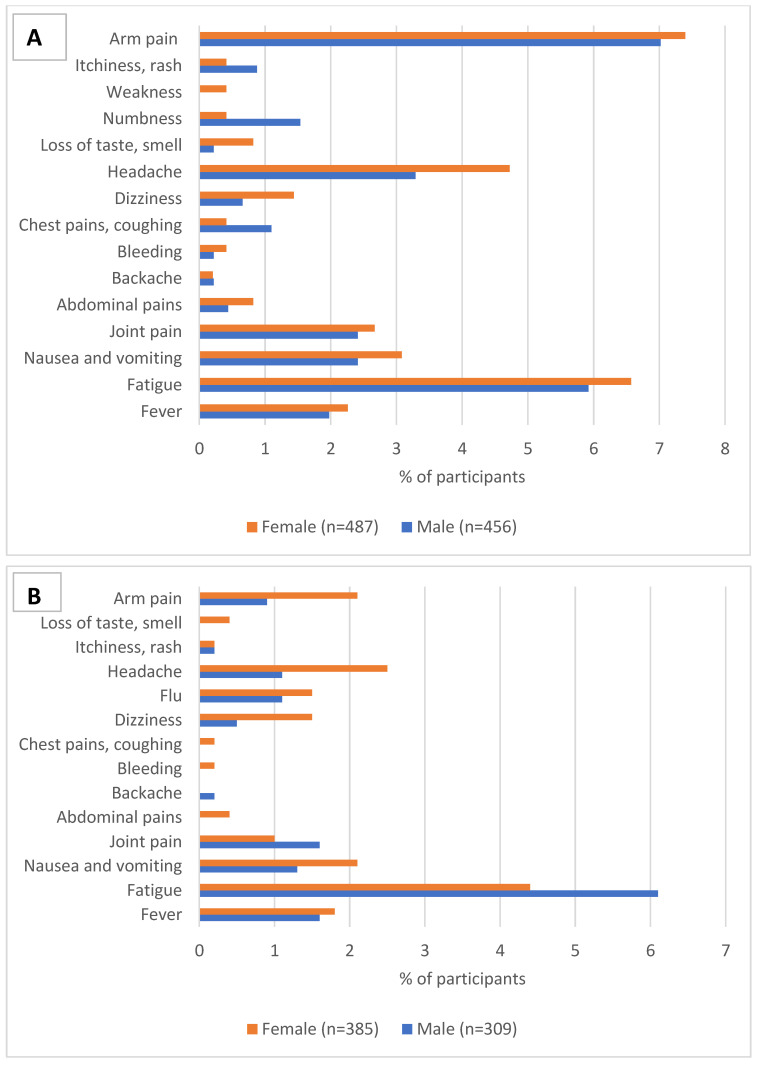
Frequency of vaccination adverse events by sex for the two doses (**A**) adverse events following first dose; (**B**) adverse events following second dose.

Adverse event frequency was approximately the same among the female (27.5%) and male (24.1%) participants after the first dose. There were differences in reported adverse events between female and male participants for both the first and second dose (Figure 1). Among the reported adverse events after the first dose, only numbness was significantly higher in the male compared to the female participants (6.4% and 1.5%, *p* = 0.043). The prevalence of other adverse events was not significantly different between the male and female participants: fatigue (males 5.9% vs. females 6.6%, *p* = 0.83); joint pain (males 5.9% vs. females 6.6%, *p* = 0.83); and flu-like syndrome (males 1.1% vs. females 2.1%, *p* = 0.33). No significant differences in adverse events were noted between the sexes after the second dose: fever (males 1.1% vs. females 1.5%, *p* = 0.90); joint pain (males 1.1% vs. females 0.8%, *p* = 0.32); and dizziness (males 0.5% vs. females 1.5%, *p* = 0.25).

The frequency of adverse events after the first dose was approximately similar across age groups, 82 (24.6%) for the 18–25-year-old age group, 96 (25.4%) for the 26–39-year-old age group and 66 (28.7%) for participants above 40 years (Table 4). Arm pain was the most frequently reported side effect by those aged 18–25-year (8.4%) and those aged 40+ (8.7%). For the 26–39-year-old age group, fatigue was the most frequently reported side effect (7.4%). There were significantly lower frequencies of adverse events after the second vaccination dose, 24.6% to 11.4% (*p* = 0.00008) for the 18–25-year-old age group, 25.4% to 15.6% (*p* = 0.00288) for the 26–39-year-old age group and 28.7% to 16.4% (*p* = 0.0027) for the participants aged above 40 years. There was a significant drop in frequency of participants who reported arm pain, from 28 (8.4%) after the first dose to 2 (0.8%) (*p* < 0.00001) after the second dose.

We evaluated factors associated with the presence of at least one side effect following the first and second dose. Participants who had personal COVID-19 experience were less likely to report a side effect than those with no experience (OR 0.67; 95%CI: 0.49–0.90). Similarly, participants who perceived Sinovac/Sinopharm vaccines as safe (OR 0.59, 95%CI 0.44, 0.80) were less likely to report experiencing a side effect after the first dose (Table 5).

There was a trend towards increased likelihood of reporting one or more side effect following the second dose among those that had HIV infection compared to those without (OR 1.87; 95%CI: 1.08, 3.24, *p* = 0.026) in the univariate analysis model that did not hold in the multivariate analysis

## 4. Discussion

The SARS-CoV-2 pandemic resulted in the rapid development of effective vaccines. As of August, 2022, the WHO has approved 15 vaccines for SARS-CoV-2 across multiple platforms [15]. Approved vaccines include mRNA vaccines, viral vector vaccines, protein subunit vaccines and inactivated whole virus vaccines. Clinical trials provide important safety and efficacy data but may not always reflect real-world safety and efficacy. Whole inactivated vaccines have played an important role in the global public health response to COVID-19 and are widely used in Africa. Although there is significant literature on adverse events following vaccination with mRNA and viral vector vaccines [16,17,18,19,20] there is limited real-world data on the side effect profile following vaccination with inactivated vaccines in Africa.

Our study indicates that whole inactivated vaccines are well tolerated. Adverse events were reported by 26% of participants after the first dose. The reported frequency was lower than that reported in clinical trials where up to 44% reported adverse events [10]. The adverse events were reported as mild. A small minority of participants sought medical care for their symptoms, but no participant required hospitalization. An online survey of healthcare workers in India also found a similar frequency (24.4%) of post-vaccination symptoms among those that received the Sinopharm vaccine [21]. The symptoms were mild and of short duration. In contrast, the frequency of adverse events that we observed is lower than that from an online survey conducted in the UAE among Sinopharm vaccine recipients where 75.6% reported at least one adverse events [11].

We observed a lower frequency of adverse events reported after the second dose compared with the first dose. This finding contrasts with other vaccine platforms, such as mRNA vaccines where in clinical trials and real-world data, the second dose appears to be more reactogenic than the first dose [17,18,22].

The primary local reaction was pain in the injection-site arm. This was reported by 68 (7%) and 14 (2%) participants following the first and second dose. The frequency of arm pain significantly diminished between the first and the second doses. Pain at the injection site was also the most frequently cited local side effect in the clinical trials [10].

Systemic adverse events were similar in nature though lower in frequency to those reported in the clinical trials. Fatigue and headache were the most prominent adverse events. In a phase 3 clinical trial with the Sinopharm vaccine, headache occurred in up to 13.1% and fatigue in about 11% of the participants [10]. The frequency of headache was 4.1% and fatigue 6.3% following the first dose in this study. The frequency of these adverse events was lower after the second dose. Headache was the main symptom that led many individuals to seek medical attention. The other commonly reported side effect was nausea and vomiting (2.8% and 1.7% after the first and second doses, respectively), as well as joint pains (2.6% and 1.3% after first and second doses, respectively). The frequency of these adverse events was comparable to those reported in the Phase 3 clinical trial where nausea and vomiting were reported in 1.6%, arthralgia in 1.4% and myalgia in 5.4% [10].

Many studies have typically noted a higher frequency of adverse events in women than men. These have often been online surveys or surveys of healthcare workers with higher engagement of female respondents who have mostly received mRNA vaccines [23,24]. Our study was generally balanced in sex and did not note a significant difference in the frequency of adverse events between men and women. The exception was with ‘numbness’ which was reported at a higher frequency in men than women. The difference in our data may be due to the relatively low frequency of adverse events from the inactivated vaccines that we observed compared with other vaccine types that noted a higher frequency of adverse events among women [25].

We did not note a significant difference in reported adverse events based on age. This is in contrast to other vaccine studies that have noted age-related differences in the frequency of reported side effects [17,21]. Due to the demographic profile in Zimbabwe, we had a very low frequency of individuals above the age of 60 years within the study and a cut off of 40 years may not be sensitive enough to identify significant age-related differences.

In our study, 33 (3.5%) participants reported missing work after the first vaccine dose, with most missing 1–2 days. The number of participants reporting missed days dropped after the second dose to 4 (0.6%) participants. A US study of healthcare workers who mostly received mRNA vaccines noted that 4.1% of vaccinations resulted in short-term disability claims of 1–3 days for missed work [26]. Claims rates were higher for younger workers and those in office positions compared to clinical care staff. In contrast, the claims were higher after the second dose compared with the first dose [26]. Similar to other published studies, our data suggest that the amount of missed work following vaccination is low. However, each day of missed work, particularly for those that are self-employed and largely in the informal sector, may have significant financial impact on individuals and their families.

A well-described phenomenon in clinical care and research is the placebo and nocebo effect that drive positive and negative expectations in outcomes [27]. Psychosocial factors can play an important role in influencing our attitudes towards a therapy. We observed an association between reporting of adverse events and perceived vaccine safety and personal experience with COVID-19. Individuals who reported that they perceived the Sinopharm/Sinovac vaccines as safe at their baseline interview were less likely to report a vaccine side effect. The baseline interview was conducted within a few minutes after the first vaccine dose. Similarly, those who reported that they knew someone who had been seriously ill or died from COVID-19 were less likely to report a side effect. A recent study in the United States found that pre-vaccine side effect expectations, worry about COVID-19 and depressive symptoms predicted reported COVID-19 vaccine adverse events [28]. This is consistent with our findings showing that psychological factors may influence reported adverse events.

Most of the study participants were vaccinated with the Sinopharm vaccine, and only three participants received the CoronaVac vaccine. The CoronaVac vaccine in Phase 1/2 trials reported an adverse event frequency of up to 35% compared with 22% in the alum placebo group, the adverse events were mild and typically resolved within 48 h [29]. The most common adverse events were injection-site pain in up to 26% of participants. The findings following CoronaVac vaccination were similar to those following Sinopharm vaccination [9,29]. We did not disaggregate or exclude participants based on vaccination type.

One of the strengths of our study is that the cohort was prospectively followed from the first dose through to the second dose. The prospective nature of our study enabled us to follow up all of the participants within a pre-defined time following vaccination. This contrasts with several studies that provide real-world evidence for post-vaccination symptoms using online surveys where the survey is conducted at variable time-points following vaccination. Although the concern for recall bias remains in our study design, we anticipate that this is less significant than online surveys where recall bias is coupled with the self-selection of participants and a variable time between vaccine dose and survey completion.

Another key component of our study is that it is one of the only studies that provides data on inactivated whole vaccine safety in an African cohort. These data will be informative to public health practitioners as they convey the risks and benefits of the vaccines to African populations.

## 5. Limitations

The main limitations of our study are related to sample size and recall. The sample size was based on a survey to evaluate attitudes to vaccination and determine adverse events to vaccination. We recruited 1016 participants at baseline. The detection of true adverse events following vaccination typically occurs over millions of doses. Several case reports and case series have provided important information on COVID-19 vaccine adverse events. Reports are available for several vaccine platforms, but these reports often come from settings with robust healthcare infrastructure for pharmacovigilance and diagnostic evaluation of adverse events following immunization (AEFI) reporting. AEFI reporting in Africa is limited and generally poor. Strengthening AE reporting capabilities should be a critical component of the strengthening of health systems in Africa. Our sample size is too small to provide a generalization of AEFI with inactivated vaccines. However, our data are consistent with other studies that suggest that inactivated vaccines are well tolerated with a relatively lower frequency of adverse events [21,25].

The second limitation is that the participants were asked to recall their symptoms rather than noting them in a symptom diary as would occur in a clinical trial. The recall bias may have contributed to the lower frequency of adverse events reported in this study as participants were typically followed up a median of 12 days IQR (10–16) days after their vaccination dose.

The third limitation is the inability to generalize study findings. The sample consisted of individuals presenting for vaccination with prospective follow-up following vaccination in Harare, Zimbabwe. The participants were enrolled sequentially to participate in the survey, and although equally representative of male and female participants, the population is exclusively an urban population. The education and HIV status characteristics are close to those of the national distribution [30,31]. However, the economic classifications are likely most representative of urban settings. Despite these challenges for generalizability, the data still provide some insights into adverse events as reported in a real-world urban setting in a large African city. This study has provided real-world evidence of the safety and tolerability of whole inactivated vaccines in African populations. We have previously demonstrated that vaccine hesitancy in Zimbabwe may be driven by safety concerns [14]. The type of data that we have generated in this study can be used to address those concerns and enable effective communication strategies to support vaccine effectiveness.

## 6. Conclusions

Whole inactivated vaccines against COVID-19 are safe and well tolerated. Reported side effects were mild and self-resolved. No side effects resulted in hospitalizations or deaths. The frequency of adverse events decreased after the second dose compared to after the first dose, and there were no significant differences in the frequency of reported adverse events based on sex and age. The side effects resulted in a low frequency of lost days of work. Psychosocial factors influenced side effect reporting. Individuals who had known someone who had been ill or died from COVID-19 and those who perceived vaccines as safe were less likely to report side effects than those who did not.

## Figures and Tables

**Table 1:** Survey questions addressing adverse events.

Question Text	Response Options
After the first dose of the vaccine, how was the injection site?	Little to no swelling or redness; Swelling and/or redness with pain, but no restriction in movement; Severe swelling with pain and difficulty moving
After the first dose of the vaccine, did you experience any of the following symptoms?	
Fever	Yes; No
Fatigue	Yes; No
Nausea and vomiting	Yes; No
Joint pain	Yes; No
Other (specify)	Yes; No
What was the highest temperature reading?	37.5–38.0 ℃; 38.1–38.5 ℃; 38.6–39.0 ℃; 39.1 ℃ or over; Do not know (Do not read)
How long after receiving the dose did the fever start?	Within 12 h; Between 12 to 24 h; More than 24 h
How long did the fever last?	About 24 h; About 36 h; About 48 h; About 60 h; 72 h or more; Do not know
Did you miss any days of work because of adverse events from the vaccine?	Interviewer recorded the number of days
Did you have any post-vaccination symptoms that required you to see a medical professional?	Yes; No
What were the symptoms? Select all that apply.	Fever or chills; Cough; Shortness of breath or difficulty breathing; Fatigue; Muscle or body aches; Headaches; New loss of taste or smell; Sore throat; Congestion or runny nose; Nausea or vomiting; Diarrhea; Other
In general, COVID-19 vaccines are safe	Strongly agree, Somewhat agree, Neutral, Somewhat disagree, Strongly disagree, Do not know, Prefer not to answer
I am confident that my country’s regulation process approved the COVID-19 vaccine, only when it was shown to be safe	Strongly agree, Somewhat agree, Neutral, Somewhat disagree, Strongly disagree, Do not know, Prefer not to answer
I am confident that COVID-19 vaccines are effective in preventing the disease	Strongly agree, Somewhat agree, Neutral, Somewhat disagree, Strongly disagree, Do not know, Prefer not to answer
How safe or unsafe is the Sinopharm/*Sinovac* vaccine?	Very safe, Somewhat safe, Somewhat unsafe, Very unsafe, Do not know, Prefer not to answer
Do you personally know anyone who became seriously ill or died as a result of COVID-19?	I do not personally know anyone who became seriously ill or died because of COVID-19, Spouse or parent, Sibling, Child, Friend, Neighbor, Co-worker, Other
Has a doctor or health professional ever told you that you have any of the following conditions?	Diabetes, Cardiac disease, Respiratory illness, HIV, Hypertension/high blood pressure, None of the above

**Table 2:** Baseline characteristics of the study participants.

Characteristics	Baseline (n = 1016)	Follow Up after First Dose (n = 943)	Follow Up after Second Dose (n = 922)
Gender (Female)	508 (50%)	487 (51.6%)	482 (52.3%)
Median age (IQR)	30 (22–39)	30 (23–39)	30 (23–39)
Age groups			
18–25	368 (36.2%)	334 (35.4%)	326 (35.4%)
26–39	409 (40.3%)	379 (40.2%)	371 (40.2%)
≥40	239 (23.5%)	230 (24.4%)	225 (24.4%)
Ethnicity (Black African)	1016 (100%)	943 (100.0%)	922 (100.0%)
Co-morbid conditions			
Diabetes	15 (1.5%)	14 (1.5%)	13 (1.4%)
Cardiac Disease	4 (0.4%)	4 (0.4%)	4 (0.4%)
Respiratory Illness	23 (2.3%)	23 ((2.4%)	21 (2.3%)
Hypertension	67 (6.6%)	63 (6.7%)	62 (6.7%)
HIV	126 (12.4%)	119 (12.6%)	117 (12.7%)
Know someone who became seriously ill or died as a result of COVID-19	428 (42.1%)	393 (41.7%)	384 (41.6%)

**Table 3:** Prevalence of adverse events after the first and second vaccination doses.

Adverse Reaction	Dose 1 (n = 938)	Dose 2 (n = 694)	*p* Value
Number of adverse events			
Have ≥1 side effect	244 (26.0%)	100 (14.4%)	<0.0001
1 side effect	202 (21.5%)	80 (11.5%)	<0.00001
2–3 adverse events	38 (4.1%)	16 (2.3%)	0.0455
4 adverse events	4 (0.4%)	4 (0.6%)	0.56868
Adverse reactions requiring medical attention	16 (1.7%)	15 (2.2%)	0.4654
Local Reactogenicity			
Little to no swelling or redness	916 (97.1%)	670 (96.5%)	0.4902
Swelling and/or redness with pain, but no restriction in movement	22 (2.3%)	22 (3.2%)	0.267
Severe swelling with pain and difficulty moving	0	2 (0.3%)	0.09296
Itchiness, rash	6 (0.6%)	2 (0.3%)	0.3843
Arm Pain	68 (7.2%)	14 (2.0%)	<0.00001
Systemic Reactogenicity			
Fever	20 (2.1%)	12 (1.7%)	0.56192
Fatigue	59 (6.3%)	36 (5.2%)	0.34722
Nausea and vomiting	26 (2.8%)	12 (1.7%)	0.1443
Joint pain	24 (2.6%)	9 (1.3%)	0.06724
Abdominal pain	6 (0.6%)	2 (0.3%)	0.3843
Backache	2 (0.2%)	1 (0.1%)	0.61708
Bleeding	3 (0.3%)	1 (0.1%)	0.38978
Chest pains, coughing	7 (0.7%)	1 (0.1%)	0.07186
Dizziness	10 (1.1%)	9 (1.3%)	0.0703
Headaches	38 (4.1%)	17 (2.5%)	0.74896
Loss of taste, smell	5 (0.5%)	2 (0.3%)	0.53526
Numbness	9 (1.0%)	0	0.0096
Weakness	2 (0.2%)	0	0.238

**Table 4:** Prevalence of adverse events after first and second vaccination doses by age group.

	First Dose Adverse Events			Second Dose Adverse Events		
	18–25 (*n =* 333)	26–39 (*n =* 378)	40+ (*n =* 230)	18–25 (*n =* 236)	26–39 (*n =* 263)	40+ (*n =* 195)
Have ≥1 side effect	82 (24.6%)	96 (25.4%)	66 (28.7%)	27 (11.4%)	41 (15.6%)	32 (16.4%)
Arm Pain	28 (8.4%)	20 (5.3%)	20 (8.7%)	2 (0.8%)	2 (0.8%)	10 (5.1%)
Fever	6 (1.8%)	10 (2.6%)	4 (1.7%)	4 (1.7%)	5 (1.9%)	3 (1.5%)
Fatigue	19 (5.7%)	28 (7.4%)	12 (5.2%)	10 (4.2%)	19 (7.2%)	7 (3.6%)
Nausea and vomiting	7 (2.1%)	12 (3.2%)	7 (3.0%)	3 (1.3%)	8 (3.0%)	1 (0.5%)
Joint pain	7 (2.1%)	13 (3.4%)	4 (1.7%)	3 (1.3%)	5 (1.9%)	1 (0.5%)
Abdominal pains	2 (0.6%)	0	4 (1.7%)	0	1 (0.4%)	1 (0.5%)
Backache	0	0	2 (0.9%)	0	0	1 (0.5%)
Bleeding	2 (0.6%)	0	1 (0.4%)	0	0	1 (0.5%)
Chest pains, coughing	0	4 (1.1%)	3 (1.3%)	0	1 (0.4%)	0
Dizziness	3 (0.9%)	4 (1.1%)	3 (1.3%)	2 (0.8%)	3 (1.1%)	4 (2.1%)
Flu-like syndrome	6 (1.8%)	8 (2.1%)	1 (0.4%)	5 (2.1%)	5 (1.9%)	2 (1.0%)
Headache	10 (3.0%)	18 (4.8%)	10 (4.3%)	4 (1.7%)	9 (3.4%)	4 (2.1%)
Itchiness, rash	0	4 (1.1%)	2 (0.9%)	0	0	2 (1.0%)
Loss of taste, smell	2 (0.6%)	2 (0.5%)	1 (0.4%)	0	2 (0.8%)	0
Numbness	3 (0.9%)	3 (0.8%)	3 (1.3%)	0	0	0
Weakness	2 (0.6%)	0	0	0	0	0

**Table 5:** Factors associated with having at least one side effect following the first vaccine dose.

			Univariate			Multivariable		
Variables	Category	n (%)	OR	95% CI	*p*-Value	OR	95% CI	*p*-Value
Gender	Male	110 (24.1%)	1					
	Female	134 (27.6%)	1.201	0.896, 1.609	0.22			
Age	18–25	82 (24.6%)	1					
	26–39	96 (25.4%)	1.042	0.741, 1.464	0.813			
	≥40 years	66 (28.7%)	1.232	0.843, 1.8	0.281			
Education	None + primary	19 (22.9%)	1					
	Lower secondary	167 (24.6%)	1.092	0.636, 1.876	0.75			
	Higher secondary	35 (31.3%)	1.531	0.8, 2.931	0.199			
	Tertiary	24 (35.8%)	1.88	0.919, 3.844	0.084			
Economic status	High	41 (24.7%)	1					
	Middle	138 (25.0%)	0.977	0.655, 1.457	0.909			
	Low	66 (29.5%)	1.207	0.767, 1.9	0.416			
Personal COVID Experience	No	161 (29.3%)	1			1		
	Yes	84 (21.5%)	0.667	0.492, 0.903	0.009	0.657	0.484, 0.892	0.007
HIV	Negative	211 (25.7%)	1					
	Positive	34 (28.6%)	1.222	0.799, 1.868	0.354			
In general, COVID-19 vaccines are safe	Disagree	51 (33.8%)	1					
	Agree	194 (24.6%)	0.658	0.452, 0.957	0.029			
Robust Regulatory process	Disagree	46 (27.7%)	1					
	Agree	199 (25.7%)	0.929	0.636, 1.356	0.703			
Vaccines are effective	Disagree	41 (29.7%)	1					
	Agree	204 (25.4%)	0.8	0.537, 1.192	0.273			
Safety of Sinovac/Sinopharm vaccine	Unsafe	105 (33.0%)	1			1		
	Safe	140 (22.5%)	0.596	0.442, 0.805	0.001	0.589	0.436, 0.797	0.001

## Data Availability

The data presented in this publication will be openly available in a repository with DOI number at the time of publication.
